# Lipid Levels and Atherogenic Indices as Important Predictive Parameters in the Assessment of Cardiovascular Risk in Patients with Pulmonary Tuberculosis—Slovak Pilot Study

**DOI:** 10.3390/medicina61030365

**Published:** 2025-02-20

**Authors:** Karolína Kubalová, Igor Porvazník, Mária Majherová, Lenka Demková, Anna Piotrowska, Marta Mydlárová Blaščáková

**Affiliations:** 1 Department of Biology, Faculty of Humanities and Naturel Sciences, University of Prešov in Prešov, Ul. 17 Novembra 1, 080 01 Prešov, Slovakia; karolina.kubalova@smail.unipo.sk; 2 Department of Laboratory Methods in Healthcare, Faculty of Health Science, Catholic University in Ružomberok, 60, Námestie Adreja Hlinku 1159, 034 01 Ružomberok, Slovakia; igor.porvaznik@ku.sk; 3 Department of Physics, Mathematics and Technology, Faculty of Humanities and Natural Sciences, University of Prešov in Prešov, Ul. 17 Novembra 1, 080 01 Prešov, Slovakia; maria.majherova@unipo.sk; 4 Department of Ecology, Faculty of Humanities and Natural Sciences, University of Prešov in Prešov, Ul. 17 Novembra 1, 080 01 Prešov, Slovakia; lenka.demkova@unipo.sk; 5 Institute for Basic Sciences, Faculty of Physiotherapy, University of Physical Culture, 31-571 Krakow, Poland

**Keywords:** atherogenic indices, cardiovascular risk, Castelli’s risk indices—I, II, glucose, lipids, prediction, tuberculosis

## Abstract

*Background and Objective*: Tuberculosis is one of the globally prevalent infectious diseases. Lipids play a crucial role in its development as well as in other diseases of the cardiovascular system. Cardiovascular diseases significantly worsen the functional and vital prognosis of tuberculosis patients. The aim of the study was to assess the differences in lipid profile, glucose, and atherogenic markers between tuberculosis patients and healthy individuals. *Materials and Methods*: The project involved 34 patients diagnosed with pulmonary tuberculosis (TB) and a control group (CG: n = 35). The following were assessed: total cholesterol (CHOL), low-density lipoprotein (LDL), high-density lipoprotein (HDL), triglycerides (TG), and glucose. Atherogenic indices: Castelli risk index I (CRI-I), Castelli risk index II (CRI-II), atherogenic index of plasma (AIP) and atherogenic coefficient (AC) were calculated from lipid profile parameters using appropriate formulas. *Results*: A statistically significant difference was found between CG and TB in the parameters CHOL, LDL and HDL (*p* < 0.001). Based on the calculated atherogenic indices CRI-I and AIP, people diagnosed with TB can be classified into the high cardiovascular risk group. By fitting the ROC curve, atherogenic indices were shown to be effective predictors of cardiovascular risk in people with tuberculosis. *Conclusions*: Atherogenic indices are useful markers for detecting cardiovascular disease in patients with tuberculosis and may help identify cardiovascular risks that might otherwise be missed.

## 1. Introduction

Tuberculosis is an infectious disease caused by bacteria (*Mycobacterium tuberculosis*). While the disease typically affects the lungs (pulmonary tuberculosis), it can also impact various organ systems, including the lymphatic system, central nervous system, gastrointestinal system, and cardiovascular system [1,2]. The disease carries high morbidity and mortality. Typical symptoms associated with tuberculosis include persistent cough, fever, decreased appetite, weight loss, night sweats, and hemoptysis [3]. Pulmonary tuberculosis involves various pathological mechanisms, including lipid peroxidation and antioxidant depletion [4]. *Mycobacteria* contain various types of non-structural and structural lipids, serving as a primary energy source and playing a crucial role in the pathogenicity, virulence, and persistence of *mycobacteria* [5]. Wilburn et al. [6] state that lipids play a key role not only in the development of tuberculosis but also in cardiovascular diseases. Cholesterol constitutes approximately one-third of the lipid content of the cell membrane and plays a vital role in several metabolic pathways, such as anaplerotic assimilation through the methylcitrate cycle, anaplerotic assimilation via the methylmalonyl pathway, and biosynthetic incorporation into methyl-branched polyketide lipids [6,7,8].

Modified lipid metabolism may influence susceptibility to tuberculosis. Contradictory studies [9,10] have been published regarding the relationship between total cholesterol (CHOL), low-density lipoprotein (LDL), and high-density lipoprotein (HDL) levels and the risk of developing tuberculosis. Risks associated with low blood cholesterol levels include hypocholesterolemia, which is linked to an increased risk of tuberculosis. Hypercholesterolemia has the opposite effect in relation to tuberculosis [10]. Lower serum levels of LDL, HDL, and total cholesterol correlate with more extensive lung disease [11]. Cholesterol levels and the risk of tuberculosis are influenced by several factors, such as body mass index (BMI), diabetes mellitus (DM), and statin use, complicating the understanding of their relationship [12].

Patients with tuberculosis have an increased incidence of cardiovascular and cerebrovascular diseases [11]. Cardiovascular complications of tuberculosis are among the most common extrapulmonary manifestations of the disease [13]. Tuberculosis and cardiovascular diseases closely overlap epidemiologically and pathogenetically. Stratification of cardiovascular risk is essential for improving preventive therapeutic interventions. Biomarkers are an integral part of predictive models when identifying risk groups of individuals.

In recent years, several new specific biomarkers reflecting the risk of cardiovascular diseases have been identified and studied, such as the atherogenic index of plasma (AIP), Castelli’s risk index I and II (CRI-I and II), and the atherogenic coefficient (AC) [14]. The AIP is derived from the logarithmic transformation of the triglyceride to HDL ratio and is considered a significant indicator of cardiometabolic health [15]. Javardi et al. [16] state that it is an optimal indicator of dyslipidemia and related disorders. Castelli’s risk index I, also known as the cardiac risk factor, reflects the formation of coronary plaques. Castelli’s risk index II is a predictor of cardiovascular risk concerning the triglycerides (TG)/HDL-C (cholesterol) ratio, correlating with insulin resistance and predicting acute myocardial infarction [17].

The aim of the study was to assess the differences in the concentrations of lipid profile markers, glucose, and atherogenic indices between patients with tuberculosis (TB) and healthy individuals.

## 2. Materials and Methods

The study sample consisted of 34 patients diagnosed with tuberculosis, including 24 men and 10 women. The control group consisted of 35 people, including 21 men and 14 women. The mean age in the group of patients with tuberculosis was 49.85 ± 16.04 years, while the mean age in the control group was 44.63 ± 14.34 years. The study was conducted from March 2022 to March 2023. Samples were provided by the National Institute of Tuberculosis, Pulmonology and Thoracic Surgery in Vyšné Hágy (Slovak Republic). The inclusion criteria for study participants included people with diagnosed pulmonary tuberculosis confirmed by sputum examination, regardless of gender, aged 18 years and older. Exclusion criteria included individuals with retroviral diseases, kidney diseases, neoplastic diseases, pregnant and breastfeeding women, obesity, diabetes, hypertension, and patients with endocrine disorders or patients taking lipid-lowering therapy (e.g., statins). The study was conducted after obtaining written informed consent from all individuals and in accordance with the ethical principles of the Helsinki Declaration. The study protocol was approved by the Ethics Committee of the University of Prešov.

### 2.1. Biochemical Analysis

For the biochemical analysis, peripheral blood samples were collected from all individuals participating in the study. Blood was collected into SARSTEDT tubes with a volume of 5.5 mL, containing a clot activator. Using a centrifuge (MIKRO 200R, Hettich, Tuttlingen, Germany) at 4000 rpm for 10 min, the serum was separated from the blood samples. The separated serum was then transferred into Eppendorf-type tubes. The samples were analyzed using the Cobas Integra 400 plus biochemical analyzer (Roche, Basel, Switzerland). In the blood serum, selected markers of the lipid profile, including CHOL, HDL, LDL, and TG, were determined, and in plasma, the glucose (GLU) levels were measured.

### 2.2. Calculation of Atherogenic Indices

Based on the results of the lipid profile, specific biomarkers and atherogenic indices were calculated using the following formulas:

CRI-I = TC (total cholesterol)/HDL-C (1)

CRI-II = LDL-C/HDL-C(2)

AIP = Log10 (TG/HDL-C) (3)

AC = [(TC-HDL)/HDL] (4)

Based on reference values for AIP, individuals with tuberculosis and those in the control group were categorized into low, moderate, and high cardiovascular risk groups. Reference values for AIP were defined as follows: AIP < 0.1 is considered low risk, AIP between 0.1 and 0.24 is considered moderate risk, and AIP > 0.24 is considered high cardiovascular risk [15].

### 2.3. Statistical Analysis

The measured and calculated values were processed using Excel 2019 and Statistica ver. 12 (StatSoft, Prague, Czech Republic). For each biochemical parameter of the lipid profile, the mean and standard deviation were evaluated. Pearson’s correlation coefficient was utilized for correlation analysis. The Kruskal–Wallis test was employed for multiple comparisons of values. ROC curve analysis was performed to determine the diagnostic usefulness of the parameters (atherogenic indices). The ROC curve was performed in the SPSS program (version 29.0.2.0, IBM Corp., Armonk, NY, USA). The *p* < 0.05 value was considered statistically significant.

## 3. Results

From the results presented in Table 1, it was observed that the average cholesterol level in patients with tuberculosis was 3.22 ± 1.06 mmol/L, which was lower than the average cholesterol level in the control group individuals (4.64 ± 0.95 mmol/L). Hypercholesterolemia did not occur in patients with tuberculosis, as the average cholesterol level was within the reference range of 5.00 mmol/L. The average HDL cholesterol level in the tuberculosis patient group (0.82 ± 0.29 mmol/L) was lower than in the control group. This suggests that the average HDL cholesterol values in the patient group were lower than the reference range (men > 1.0 mmol/L, women > 1.30 mmol/L). The LDL cholesterol level in the control group (2.82 ± 0.78 mmol/L) and the patient group (1.93 ± 0.86 mmol/L) was within the reference range (LDL < 4.10 mmol/L). The glucose level in the tuberculosis patient group was higher (5.93 ± 3.62 mmol/L) than the reference range of 3.3–5.5 mmol/L. The Student’s *t*-test revealed a statistically significant difference in GLU (*p* < 0.024), CHOL, LDL, and HDL (*p* < 0.001).

Based on the results presented in Table 1, the average values of atherogenic indices were higher in patients with tuberculosis than in the control group. According to the results, patients with tuberculosis fall into the high cardiovascular risk group, as the average values of the atherogenic index of plasma were higher than 0.21. The Student’s *t*-test revealed a statistically significant difference in CRI-I and AC parameters (*p* < 0.020), and AIP (*p* < 0.001).

According to the Pearson’s correlation coefficient results presented in Table 2, a statistically significant association (*p* < 0.05) between atherogenic index parameters and lipid profile parameters was found in the tuberculosis patient group. Based on these results, it is concluded that associations between HDL, TG, LDL, and CHOL need to be monitored in individuals with tuberculosis, as these parameters are associated with high cardiovascular risk. Results in Table 2 show a statistically significant negative correlation between atherogenic indices and HDL in the control group. A statistically significant positive correlation was found between atherogenic indices, LDL, and triglycerides. A negative correlation was found between atherogenic indices and GLU, and a positive correlation was found between atherogenic indices and CHOL, but no statistically significant correlations between GLU and CHOL were found in the control group.

Results in Table 3 indicate a positive correlation (*p* < 0.05) between atherogenic indices in the tuberculosis patient group. In the control groups, there was mostly a positive correlation between atherogenic indices.

Table 4 shows the diagnostic utility of atherogenic indices for cardiometabolic risk in tuberculosis patients compared with individuals in the control group. Based on statistical analysis via the ROC curve, it can be concluded that in the Slovak population, the atherogenic indices.

**Figure 1:**
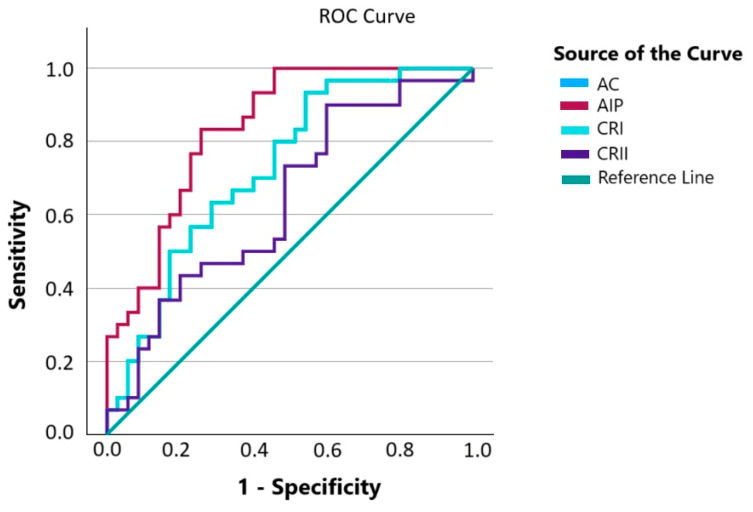
ROC curve—comparison of pulmonary tuberculosis patients with cardiometabolic risk compared to healthy individuals. AC, atherogenic coefficient. AIP; atherogenic index of plasma. CRI-I, Castelli’s risk index I. CRI-II, Castelli’s risk index II.

CRI-I and AC (0.728) are appropriately chosen indices, and AIP (0.839) is a very good index for detecting the presence of cardiovascular disease in individuals with TB (Figure 1).

## 4. Discussion

Tuberculosis ranks among the 13 most common causes of death worldwide and is the second most prevalent infectious disease after COVID-19 (Coronavirus disease 2019) [18,19]. Over the past decades, the burden of TB has been decreasing. With the COVID-19 pandemic and current political conflicts (war in Ukraine), the incidence of TB in the territory of the Slovak Republic has slightly increased. The epidemiology of tuberculosis is influenced by clinical, demographic, geographic, and social factors at the local or national level. This study was conducted to assess the differences in the concentration of the lipid profile (CHOL, HDL, LDL, and TRIGL), glucose, and atherogenic indices between the control group of individuals and those diagnosed with pulmonary TB. The relationships between the values of biochemical parameters and atherogenic indices in the above-mentioned groups of people were also assessed. Some authors [20,21] have pointed out that individuals diagnosed with TB have lower serum cholesterol levels compared to uninfected individuals. Lower levels of the lipid profile are associated with increased inflammation in patients with pneumonia, sepsis, and surgery [10,22,23]. Vitoria et al. [21] reported that lower serum levels of CHOL, HDL, and LDL were associated with a positive sputum culture for Mycobacterium tuberculosis. From the results of this study, it can be inferred that patients with pulmonary tuberculosis had average values of HDL cholesterol (0.82 ± 0.29 mmol/L) lower than the reference range for men and women (HDL men > 1.0 mmol/L, women > 1.3 mmol/L). The average value of LDL cholesterol in the control group (2.82 ± 0.78 mmol/L) and in the group of TB patients (1.93 ± 0.86 mmol/L) was within the reference range (LDL < 4.10 mmol/L). Similarly, the average level of CHOL in the TB group was lower than in the control group (CG: 4.64 ± 0.95 mmol/L; TB: 3.22 ± 1.06 mmol/L). There was no statistically significant difference between the control group and the TB group in the TG parameter (*p* < 0.564). Based on the lipid profile results, it can be indicated that patients with pulmonary tuberculosis did not experience hypercholesterolemia or hypocholesterolemia. Similar results were reported by Salunkhe et al. [24] on the Indian population, Apkovi et al. [9] on the Benin population and Air-homwanbor et al. [25] on the Nigerian population. Metwally and Raheem [26], in their study conducted on the Egyptian population, reported significantly lower values of cholesterol (*p* < 0.05) and TG (*p* < 0.01) in patients diagnosed with TB compared to the control group. In the current study, however, a higher mean TG value was obtained in patients with TB (1.19 ± 0.51 mmol/L). However, no statistically significant difference was confirmed between the control group and the group of people diagnosed with tuberculosis in the TG parameter in this study (*p* = 0.564). The different results could have been influenced by the studied population, possible comorbidities, or the immune system of the people. Mani et al. [2] in a study conducted on the Indian population reported that in patients diagnosed with tuberculosis, the value of total cholesterol was 19.25 mg/dL, while in the control group, it was 186.50 mg/dl. The present results regarding the Slovak population are similar, as the level of total cholesterol was higher in the control group (4.64 ± 0.95 mmol/L) than in patients diagnosed with tuberculosis (3.22 ± 1.06 mmol/L). Mani et al. [2] found a significant correlation between a higher prevalence of hypolipidemia in patients with pulmonary tuberculosis and healthy individuals. Patients with low lipid levels experience more severe inflammation than those with normal lipid levels. Several inverse studies [12] have been published on the relationship between lipid profile markers and the risk of developing TB. Some studies [10,12] suggest that a low cholesterol level is associated with an increased risk of TB. Low concentration of lipid profile markers in individuals diagnosed with pulmonary tuberculosis often correlates with the severity of the disease and impaired immune system function [20,27]. In contrast, Apkovi et al. [9] and Grebemicael et al. [10] suggested that low cholesterol levels in patients with TB may only be a consequence of the disease because TB treatment significantly increases low cholesterol levels. Mechanisms addressing the relationship between low cholesterol levels and the risk of developing TB are not completely understood. Innate and cell-mediated immune responses are crucial in the defense against TB [28]. Another aspect is that cholesterol levels and the risk of developing TB can be influenced by the following factors: BMI, diabetes mellitus status, and statin use, which subsequently complicates the understanding of the relationship between them [12].

Cholesterol is one of the main sources of carbon during the infection process of *Mycobacterium tuberculosis* (Mtb) and is a critical factor for optimal growth and persistence of mycobacteria. Mtb can utilize cholesterol as the sole carbon source for energy synthesis and membrane lipid production, allowing the formation of virulent lipids in its cell wall. Excessive cholesterol accumulation forms lipid droplet structures that promote the formation of foamy macrophages. The formation of foamy macrophages suppresses the host cell immune response, helping Mycobacterium tuberculosis achieve immune escape and sustained survival, leading to the development of tuberculous granulomas, tissue cavitation, and systemic spread of Mtb [29]. Cholesterol, which is found in the cell membrane of lymphocytes, is important for their cytotoxic function. Activated CD4+, CD8+, and T cell subsets of lymphocytes recruit macrophages and release molecules such as interferon and tumor necrosis factor (TNF), making them more effective against Mycobacterium tuberculosis. Due to the low cholesterol content of the cell membrane, the ability of macrophages to uptake Mtb is reduced [10]. Preclinical studies [11] have revealed an increase in secretory phospholipase A2 and serum amyloid 2 in response to tuberculosis-induced inflammation, thereby lowering HDL cholesterol levels. In addition, reverse cholesterol transport mediated by the ATP-binding cassette transporter and lecithin-cholesterol acyltransferase is reduced during inflammation, leading to a decrease in HDL. Lower serum lipid levels are also associated with higher levels of systemic inflammation in other infections [11].

The association between type 2 diabetes mellitus and TB is well known because immune-metabolic changes in diabetes cause increased susceptibility to tuberculosis [30]. Patients with tuberculosis generally experience temporary hyperglycemic states. The occurrence of hyperglycemia can be associated with stress, prolonged inflammation, changes in lipid and glucose metabolism, or insulin resistance syndrome [31,32]. In this study, it was found that individuals with pulmonary tuberculosis had a higher average fasting plasma glucose level (5.93 ± 3.62 mmol/L) than individuals in the control group (4.40 ± 1.46 mmol/L). In this biochemical parameter, a statistically significant difference (*p* = 0.024) was found between the studied groups using the Student’s *t*-test. Detection of the glycemic status of patients undergoing tuberculosis treatment is important because early detection and treatment of type 2 diabetes can improve the results of tuberculosis treatment [33]. Based on the results, it was found that 23.53% of individuals in the TB group had a glucose level > 5.6 mmol/L. Similar results were found by Tahir et al. [34] on the Pakistani population. Individuals diagnosed with TB and type 2 diabetes mellitus are predisposed to infection relapses, treatment failure, increased mortality, and delayed mycobacterial clearance [35]. Impaired glucose tolerance is an indicator of a future high risk of DM. Therefore, health counseling should be provided regarding a healthy diet, increasing physical activity, avoiding harmful alcohol consumption, and avoiding smoking to prevent the onset of type 2 diabetes mellitus [33,36].

Multisystem involvement of TB affects the cardiovascular system in various forms. The most common risk factors associated with the development of cardiovascular diseases (CVD) include conditions such as hypertension, DM, obesity, hyperlipidemia, and smoking. Risk factor analysis is important in designing strategies for the prevention of cardiovascular diseases. Monocytes, macrophages, cytokines, and lymphocytes that propagate cell-mediated responses against Mycobacterium tuberculosis are important factors controlling atherogenesis. With persistent systemic inflammation, atherosclerotic plaque formation occurs in blood vessels. Other critical components in the pathophysiology of cardiovascular complications in tuberculosis are molecular mimicry and autoimmunity, which include the heat shock protein (HSP) system. Approximately 40% to 50% of the residues in human HSP65 and Mtb HSP65 (mycobacterial heat shock protein-65) are identical. Exposition to infection induces the expression of HSP65 on the surface of endothelial cells, resulting in a cross-reaction between antibodies produced against Mtb HSP65 and the host’s own HSP60 antigen, leading to endothelial damage and stimulation of atherogenesis, a process that can result in coronary artery disease [13,37]. Cardiovascular complications associated with tuberculosis may include pericarditis. Approximately 1–2% of patients with TB have associated pericarditis. This type of pericarditis is characterized by a significant inflammatory state and a high risk of progression to the constrictive form [37,38].

Clinicians and researchers widely use lipid profiles as well as atherogenic indices as predictors of the risk of developing atherosclerotic cardiovascular diseases [39]. Castelli’s risk indices (CRI-I and CRI-II) are two important indicators of vascular risk whose predictive value is greater than isolated lipid parameters [40]. The most suitable index for tracking cardiovascular risk is the logarithmically transformed molar ratio of triglycerides to HDL cholesterol. This study investigated the atherogenic index of plasma (AIP) using the formula Log10/(TRIGL/HDL-C). Apkovi et al. [9], in their research on individuals in West Africa, found a significantly increased atherogenic index in patients with TB without reaching a critical level, which is synonymous with atherogenic risk. Atherogenic indices, including Castelli’s risk index I (CRI-I 4.55 ± 0.28), Castelli’s risk index II (CRI-II 2.89 ± 0.23), and AIP (0.41 ± 0.04), were elevated in patients with the highest range of smear positivity. In the study, individuals with TB had lower values of CRI-I (4.21 ± 1.59) and CRI-II (2.54 ± 1.33) compared to the study by Apkovi et al. [9], and conversely, higher values of AIP (0.51 ± 0.23). Based on the AIP value, both research groups of individuals with TB can be classified into the category of high cardiovascular risk (AIP > 0.24). Markelić et al. [41], in the Croatian population, found lower values of atherogenic indices in patients with chronic obstructive pulmonary disease (AIP −0.177 ± 0.283; CRI-I 3.29; CRI-II 1.99) compared to the control group (AIP −0.005 ± 0.269; CRI-I 4.15; CRI-II 2.66). In this study, higher average values of atherogenic indices in the group of individuals with TB were observed. A statistically significant difference between the control group and individuals with -TB was found in the parameters CRI-I and AIP (*p* = 0.020) and AIP (*p* < 0.001). Bhardwaj et al. [42] suggest that lipid ratios like AIP, CRI, and the atherogenic coefficient can be used to identify individuals with a higher risk of cardiovascular diseases. Castelli’s risk index I (CRI-I), also known as the cardiac risk ratio, reflects the formation of coronary plaques [17]. A CRI-I value < 3.5 is considered a normal value. A CRI-I value is >3.5 is considered a high cardiovascular risk. In the study, individuals with TB have a high cardiovascular risk based on the CRI-I value (4.21). A CRI-II value < 3.0 is considered a normal value. A CRI-II value (>3.0) indicates a high risk of cardiovascular disease [43]. Threshold values for classifying individuals into high cardiovascular risk categories (based on AIP and CRI-I) were determined based on selected studies [44,45].

The average values of CRI-II in the study were in the normal range in the group of patients with TB (2.54 ± 1.33) and the control group (2.15 ± 0.99).

Based on the evaluation using the ROC curve as a tool showing the relationship between specificity and sensitivity, it was found that the atherogenic indices CRI-I, AC, and AIP are appropriate and reasonable indicators (AUC: CRI-I and AC 0.728; AIP 0.839) for detecting and underestimating cardiometabolic risk (Figure 1).

In this study, the interrelationships between lipid markers and atherogenic indices in people with TB were also monitored. A moderately strong positive correlation was found between TG and all atherogenic indices. Conversely, a moderately strong negative correlation was found between all atherogenic indices and HDL. No correlation was found between AIP and CHOL and AIP and LDL in the group of people with TB (Table 2). In the control group, a strong positive correlation was shown between AIP and TG (r = 0.829, *p* < 0.05) (Table 2). A statistically significant strong correlation was also shown between atherogenic indices CRI-I and CRI-II as well as between AIP and CRI-II (r = 0.972, *p* < 0.05) in the group of people with TB. In the control group, a statistically significant strong positive correlation was found between CRI-I and CRI-II (r = 0.979, *p* < 0.05), CRI-I and AIP, and AC and AIP (r = 0.801, *p* < 0.05) (Table 4).

The analysis of lipid profile markers and other pulmonary biomarkers in patients with TB at the beginning of treatment is important for better patient management and treatment outcomes. Inflammation-related lipid dysfunction should be considered instead of absolute lipid levels when predicting cardiovascular risk in patients diagnosed with TB. Cardiovascular damage significantly worsens the functional and vital prognosis of TB patients. Infectious diseases can accelerate the atherosclerotic process and also complicate cardiovascular diseases. Although patients with pulmonary tuberculosis have low lipid levels, their elevated atherogenic indices and inflammatory nature increase their probability of developing cardiovascular disease. In tuberculosis, atherogenic indices give us a more complete view of cardiovascular risk than monitoring lipid profile levels alone. The result of this study suggests that tuberculosis is a potential risk factor for cardiovascular diseases such as atherosclerosis. Cardiovascular damage significantly worsens the functional and vital prognosis of a patient diagnosed with TB. A multidisciplinary approach in conjunction with the calculation of atherogenic indices may be associated with more effective treatment in patients. Based on the results of biochemical analysis, calculated atherogenic indices and ROC curve, it can be concluded that further scientific research is needed to investigate factors influencing the lipid profile in TB patients as well as to study the impact of lipid levels on disease severity. In the future, it is necessary to consider intraindividual concentration variability of TG, LDL-C, and HDL-C levels, focus on ethnic differences, the influence of body composition as well as other influenceable and non-influenceable risk factors contributing to the development of TB. The predictive value of atherogenic indices relative to traditional models (e.g., Framingham Risk Score) remains unclear. A comparative analysis would strengthen clinical relevance.

This study has several limitations. The relatively small sample size may have reduced the statistical power of this study. The sample size is influenced by the fact that the data were collected from a single center (institute). From another perspective, the dietary habits, physical activity, and stress load of the subjects were not taken into account, which may have influenced the results of the biochemical analysis to some extent.

Regular physical activity can minimize the risk of heart disease, while also affecting blood sugar levels, blood pressure levels, and lipid levels in the body. Low socioeconomic status is associated with cardiovascular risk due to a confluence of biological, behavioral, and psychosocial risk factors that are more prevalent in disadvantaged individuals. Poor dietary choices may also contribute to increased CVD risk in people of low socioeconomic status. Biologically active parts of many food components can directly or indirectly participate in the regulation of biological metabolism. Diet is one of the main sources of nutrient intake, different food components contain different nutrients, which can lead to individual differences in cardiovascular health. A low-fat diet reduces the risk of death and myocardial infarction in people with increased cardiovascular risk. Nutrients have an irreplaceable role in the development, prevention, and treatment of CVD [46,47,48].

A pilot study can be used to assess the feasibility of recruitment, randomization, retention, evaluation procedures, and implementation of a new intervention. Components of the study that are considered unsatisfactory may be modified or removed altogether in subsequent studies. In summary, pilot studies are a necessary first step in the study of new interventions.

## 5. Conclusions

In this study, statistically significant differences were shown in the concentrations of lipoproteins (CHOL, LDL, and HDL) assessed in patients with tuberculosis and healthy individuals. Based on the calculated atherogenic CRI-I and AIP indices, the group of people diagnosed with tuberculosis can be classified into the category of high cardiovascular risk. Analysis using the ROC curve showed that CRI-I, AC, and AIP indices are better markers for early detection of cardiometabolic risk in people diagnosed with tuberculosis than lipid profile markers alone. In the studied group of patients, glucose concentration disorders were indicated. In the future, analysis of other lipid profile markers, glucose, and especially monitoring of the atherogenic indices value is needed because these are appropriate and important indices enabling detection and underestimation of cardiometabolic risk in patients with tuberculosis. Atherogenic indices are useful markers for detecting cardiovascular diseases in patients with tuberculosis and may help to identify cardiovascular risks that might otherwise be missed. The mechanism of lipid metabolism in tuberculosis should be further investigated to better understand the pathophysiology of the disease, which may lead to new therapeutic approaches. As this is a pilot study in the territory of the Slovak Republic, we plan to increase the sample size in the future, taking into account the influence of ethnicity (especially Gypsy origin), health status (disease treatment), and lifestyle factors such as dietary habits and physical activity.

## Figures and Tables

**Table 1:** Mean values of biochemical parameters and atherogenic indices in the study groups.

Parameter	Control Group (n = 35) MEAN ± SD	Patients with Tuberculosis (n = 34) MEAN ± SD	*p*
CHOL (mmol/L)	4.64 ± 0.95	3.22 ± 1.06	0.001 ***
LDL (mmol/L)	2.82 ± 0.78	1.93 ± 0.86	0.001 ***
HDL (mmol/L)	1.46 ± 0.44	0.82 ± 0.29	0.001 ***
TRIGL (mmol/L)	1.11 ± 0.52	1.19 ± 0.51	0.564
GLU (mmol/L)	4.40 ± 1.46	5.93 ± 3.62	0.024 *
CRI-I	3.43 ± 1.10	4.21 ± 1.59	0.020 *
CRI-II	2.15 ± 0.99	2.54 ± 1.33	0.171
AC	2.43 ± 1.10	3.21 ± 1.59	0.020 *
AIP	0.22 ± 0.27	0.51 ± 0.23	0.001 ***

SD, standard deviation; *p*, statistical significance value; CHOL, cholesterol; LDL, low-density lipoprotein; HDL, high-density lipoprotein; TRIGL, triglycerides; GLU, glucose; CRI-I, Castelli risk index I; CRI-II, Castelli risk index II; AC, atherogenic coefficient; AIP, atherogenic index of plasma; * *p* < 0.05, *** *p* < 0.001, statistical significance.

**Table 2:** Correlation analysis of atherogenic indices with lipid profile and glucose parameters in a group of patients with tuberculosis and in a control group of subjects.

TB patients	Parameter	GLU		TRIGL		CHOL		HDL		LDL	
		r	***p***	r	***p***	r	***p***	r	***p***	r	***p***
	CRI-I	−0.049	0.784	0.571	0.001 ***	0.502	0.002 **	−0.494	0.003 **	0.644	0.001 ***
	CRI-II	0.010	0.955	0.561	0.001 ***	0.598	0.001 ***	−0.379	0.027 *	0.751	0.001 ***
	AC	−0.049	0.784	0.571	0.001 ***	0.502	0.002 **	−0.494	0.003 **	0.644	0.001 ***
	AIP	−0.224	0.203	0.689	0.001 ***	0.170	0.337	−0.658	0.001 ***	0.272	0.120
Control group		r	***p***	r	***p***	r	***p***	r	***p***	r	***p***
	CRI-I	−0.040	0.818	0.484	0.003 **	0.185	0.287	−0.746	0.001 ***	0.550	0.001 ***
	CRI-II	−0.124	0.480	0.370	0.029 *	0.166	0.339	−0.737	0.001 ***	0.587	0.001 ***
	AC	−0.040	0.818	0.484	0.003 **	0.185	0.287	−0.746	0.001 ***	0.550	0.001 ***
	AIP	0.136	0.437	0.829	0.001 ***	0.030	0.866	−0.743	0.001 ***	0.268	0.119

*p*, statistical significance value; CHOL, cholesterol; LDL, low-density lipoprotein; HDL, high-density lipoprotein; TRIGL, triglycerides; GLU, glucose; CRI-I, Castelli risk index I; CRI-II, Castelli risk index II; AC, atherogenic coefficient; AIP, atherogenic index of plasma; * *p* < 0.05, ** *p* < 0.01, *** *p* < 0.001, statistical significance.

**Table 3:** Pearson’s correlation test between atherogenic indices in a group of patients with tuberculosis and in a control group of individuals.

TB patients	Parameter	CRI-I		CRI-II		AC		AIP	
		r	***p***	r	***p***	r	***p***	r	***p***
	CRI-I	—	—	0.972	0.001 ***	—	—	0.765	0.001 ***
	CRI-II	0.972	0.001 ***	—	—	0.972	0.001 ***	0.651	0.001 ***
	AC	—	—	0.972	0.001 ***	—	—	0.765	0.001 ***
	AIP	0.765	0.001 ***	0.651	0.001 ***	0.765	0.001 ***	—	—
Control group		r	***p***	r	***p***	r	***p***	r	***p***
	CRI-I	—	—	0.979	0.001 ***	—	—	0.801	0.001 ***
	CRI-II	0.979	0.001 ***	—	—	0.979	0.001 ***	0.717	0.001 ***
	AC	—	—	0.979	0.001 ***	—	—	0.801	0.001 ***
	AIP	0.801	0.001 ***	0.717	0.001 ***	0.801	0.001 ***	—	—

*p*, statistical significance value; CHOL, cholesterol; LDL, low-density lipoprotein; HDL, high-density lipoprotein; TRIGL, triglycerides; GLU, glucose; CRI-I, Castelli risk index I; CRI-II, Castelli risk index II; AC, atherogenic coefficient; AIP, atherogenic index of plasma; *** *p* < 0.001, statistical significance.

**Table 4:** Diagnostic utility of atherogenic indices in individuals with tuberculosis with cardiometabolic risk compared with individuals without cardiometabolic risk.

Area Under the Curve					
95% Confidence Interval					
Test Result Variable	AUC	Std. Error	Asymptotic Sig.	Lower Bound	Upper Bound
CRI-I	0.728	0.062	0.000	0.606	0.849
CRI-II	0.636	0.069	0.049	0.500	0.772
AC	0.728	0.062	0.000	0.606	0.849
AIP	0.839	0.048	0.000	0.745	0.934

AUC, the area under the curve; CRI-I, Castelli risk index I; CRI-II, Castelli risk index II; AC, atherogenic coefficient; AIP, atherogenic index of plasma.

## Data Availability

The data of this article is available upon reasonable request to the corresponding author (M.M.B.).

## Abbreviations

The following abbreviations are used in this manuscript:

AC	atherogenic coefficient
AIP	atherogenic index of plasma
AUC	the area under the curve
BMI	body mass index
C	cholesterol
CG	control group
CHOL	total cholesterol
COVID-19	Coronavirus disease 2019
CRI-I	Castelli’s risk index I
CRI-II	Castelli’s risk index II
CVD	cardiovascular diseases
DM	diabetes mellitus
GLU	glucose
HDL	high-density lipoprotein
HSP	heat shock protein
LDL	low-density lipoprotein
Mtb	*Mycobacterium tuberculosis*
Mtb HSP65	mycobacterial heat shock protein-65
*P*	statistical significance value
SD	standard deviation
TB	pulmonary tuberculosis
TG	triglycerides
TNG	tumor necrosis factor
TRIGL	triglycerides
